# Improved thermoelectric performance of solid solution Cu_4_Sn_7.5_S_16_ through isoelectronic substitution of Se for S

**DOI:** 10.1038/s41598-018-26362-z

**Published:** 2018-05-29

**Authors:** Jiaolin Cui, Tongtong He, Zhongkang Han, Xianglian Liu, Zhengliang Du

**Affiliations:** 1 School of Materials & Chemical Engineering, Ningbo University of Technology, Ningbo, 315211 China; 2 Division of Interfacial Water and Key laboratory of Interfacial Physics and Technology, Shanghai Institute of Applied Physics, Chinese Academy of Sciences, Shanghai, 201800 China

## Abstract

Cu-Sn-S family of compounds have been considered as very competitive thermoelectric candidates in recent years due to their abundance and eco-friendliness. The first-principles calculation reveals that the density of states (DOS) increases in the vicinity of the Fermi level (*E*_f_) upon an incorporation of Se in the Cu_4_Sn_7.5_S_16−*x*_Se_*x*_ (*x* = 0–2.0) system, which indicates the occurrence of resonant states. Besides, the formation of Cu(Sn)-Se network upon the occupation of Se in S site reduces the Debye temperature from 395 K for Cu_4_Sn_7_S_16_ (*x* = 0) to 180.8 K for Cu_4_Sn_7.5_S_16−*x*_Se_*x*_ (*x* = 1.0). Although the point defects mainly impact the phonon scattering, an electron-phonon interaction also bears significance in the increase in phonon scattering and the further reducion of lattice thermal conductivity at high temperatures. As a consequence, the resultant TE figure of merit (ZT) reaches 0.5 at 873 K, which is 25% higher compared to 0.4 for Cu_4_Sn_7.5_S_16_.

## Introduction

As the world’s demand for energy grows, the search for environmentally benign and earth abundant bulk thermoelectric (TE) devices is becoming increasingly important. The ternary Cu-Sn-S compounds have been paid much attention to in recent years^1–3^. Unfortunately, the TE performances of some existing Cu-Sn-S compounds are not satisfying. For example, the TE figure of merit (ZT) of Zn-doped Cu_2_SnS_3_ is 0.58 at 723 K^4^, while that of the pristine Cu_4_Sn_7_S_16_ is only 0.2 at 600 K from measurement^1^. In order to improve the TE performance of ternary Cu-Sn-S compounds, we have employed some strategies, such as the band structure engineering in Cu_4_Sn_7_S_16_^5^ and Cu_2_SnS_3_^6^, and the coordination of the Seebeck coefficient and carrier concentration in Cu_3_SnS_4_^7^. By employing the above-mentioned approaches, we have achieved remarkable improvements in TE performance, obtaining a ZT value of 0.75 for Cu_3_Sn_1.2_S_4_^7^ and 0.4 for Cu_4_Sn_7.5_S_16_^5^. Nevertheless, a great improvement of the compounds of Cu_4_Sn_7_S_16_ family is still necessary.

The previous investigations revealed that when Sn resides in the framework of Cu_4_Sn_7_S_16_, extra Sn will make the Fermi level (*E*_f_) unpin and shift towards the conduction band, thus donating the electrons and enhancing the carrier concentration (*n*_H_)^5^. However, the enhancement of *n*_H_ seems to cease as more Sn is added. This is caused by the creation of impurity levels near the middle of the bandgap, which results in a degradation of TE performance. Accordingly, it is strongly needed to build up a more stabilized electronic structure with electrically conductive network in the Cu-Sn-S compounds. Considering that a Cu-Se bond network is essential to optimize the electrical transport properties in the Cu-Sn-Se systems^8–11^, it might be feasible to build up Cu-Se slabs in the Cu-Sn-S family of compounds by adding Se. Because of the *p-d* hybridization of Cu3*d* and Se4*p* orbitals, the density of states (DOS) near the Fermi level would be localized. In this way, we would be able to improve the electrical conductivity^12^.

In addition, the atomic mass of Se (79) is much larger than that of S (32), therefore, when Se resides in S site, the presence of Cu(Sn)-Se networks would reduce the Debye temperature and sound velocity, which may have some connections with a low lattice thermal conductivity. This is because Grüneisen parameter increases as an anharmonicity of the lattice vibrational spectrum increases^13–16^. Although such investigations with respect to the hierarchical chemical bonds contributing to the low thermal conductivity are so far limited and are mostly focused on the cubic I-V-VI_2_ semiconductors^15^ and Nowotny–Juza compounds like α-MgAgSb with a non-caged structure^13^, we believe that this strategy is important for engineering the phonon transport of Cu-Sn-S compounds. In this regard, we specially substitute Se for S in the compound Cu_4_Sn_7.5_S_16_, aiming to engineer the band structure, phonon transport, and introduce lattice disorder, similar to the cases of Cu substitution for Cd^12^ in Cu_2_CdSnSe_4_ and Se for Te in n-type PbTe based solid solutions^17,18^.

## Experimental

### Sample synthesis and preparation

The mixtures, according to the formula Cu_4_Sn_7.5_S_16−*x*_Se_*x*_ (*x* = 0.5, 1.0, 1.5 and 2.0), were loaded into four different vacuum silica tubes and heated to 723 K in 4.5 h. After maintaining this temperature for 2 h, the tubes were once again heated to 1123 K within 4 h followed by holding at this temperatutre for another 4 h. Subsequently, the molten mixtures were cooled from 1123 K to 923 K within 1.5 h, and held at 923 K for 24 h. Lastly, the ingots were cooled to RT in 7 h.

After ball milling of the ingots, the dried powders were quickly sintered using spark plasma sintering apparatus (SPS−1030) at a peak temperature of 950 K and a pressure of 60 MPa. The holding time is 2 min at 950 K. After that, the sintered bulks were cooled to RT in 2 h. The final samples are 3 mm in thickness and 2.5 mm × 12 mm in cross-section for electrical property measurements. These bulk samples were obtained from the sintered blocks with a size of ϕ 20 mm × 2.5 mm. After polishing the surfaces of two sides, the coin-shaped sintered blocks with the size of ϕ 10 mm × 1.5 mm were prepared for thermal diffusivity measurements. All sintered bulks have a theoretical density (*d*) of ∼95.0%.

### Measurements

Hall coefficients (*R*_H_) were measured by using a four−probe configuration in a system (PPMS, Model−9) with a magnetic field up to ± 5 T. The Hall mobility (*μ*) and carrier concentration (*n*_H_) were subsequently determined according to the relations *μ* = |*R*_H_|*σ* and *n*_H_ =  −1/(*e R*_H_) respectively, where *e* is the electron charge. The Seebeck coefficients (*α*) and electrical conductivities (*σ*) were measured simultaneously by using a ULVAC ZEM−3 instrument system under a helium atmosphere from RT to ~900 K, with uncertainty of <6.0% for each.

The thermal conductivities were calculated based on the equation *κ* = *dλC*_*p*_, where the thermal diffusivities λ were measured by the TC−1200RH instrument under vacuum, with an uncertainty of <10%, whereas the heat capacities (*C*_*p*_) were estimated based on Dulong–Petit rule above RT. The three physical parameters (*α*, *σ*, *κ*) were finalized by taking the average values of several samples tested by the same method. The total uncertainty for ZT value was ∼18%. In addition, the heat capacities (*C*_*p*_) for the Se-incorporated sample Cu_4_Sn_7.5_S_16−*x*_Se_*x*_ (*x* = 1.0) were measured with the measurement system (PPMS, Quantum Design) in the temperature range of 2.0–153 K, and the Debye temperature (Θ_D_) was then determined using the equation^19^:

1 $${{\rm{\Theta }}}_{D}={(\frac{12{\pi }^{2}nR}{5\beta })}^{\frac{1}{3}}$$

Here, *β* was obtained from a simple Debye model^13,20^ in the low temperatures below 10 K,

2 $$\frac{{C}_{p}}{T}=\alpha +\beta {T}^{2}$$

and *n* is the number of atoms per chemical formula. The lattice contributions (*κ*_L_) were obtained by subtracting the electronic contribution (*κ*_e_) from the total *κ*, i.e., *κ*_L_ = *κ* − *κ*_e_. Here *κ*_e_ is expressed by the Wiedemann–Franz law, *κ*_e_ = *L*_0_*σT*, where *L*_0_ is the Lorenz number, estimated at 1.5 × 10^−8^ WΩK^−2^ for not fully degenerate environment of semiconductors^21^.

### Characterization

The chemical compositions of the sample (*x* = 1.0) were checked using an electron probe micro-analyzer (EPMA) (S-4800, Hitachi, Japan) with an accuracy >97%. The microstructures of the samples (*x* = 0, 1.0) have been examined using high-resolution transmission electron microscopy (HRTEM). HRTEM images were obtained at 220 kV using JEM-2010F (Field emission TEM).

The XRD patterns were collected by powder X-ray diffractometer (D8 Advance) operating at 50 kV and 40 mA with Cu Kα radiation (λ = 0.15406 nm) and a scan rate of 4° min^−1^ in the range from 10° to 120°. The lattice constants *a* and *c* were obtained from the refinement of the X-ray patterns using Jade software.

Raman spectra of three Cu_4_Sn_7.5_S_16−*x*_Se_*x*_ powders (*x* = 0, 1.0 and 2.0) were recorded at 300 K from 50 to 4000 cm^−1^ at a resolution of 0.6 cm^−1^ using an Invia-Reflex Raman spectrometer with Nd:YAG laser source (*l* = 532.0 nm). The spectrum of the sample Cu_4_Sn_7.5_S_16_ was presented for comparison. A 50× objective lens was employed via a confocal geometry to transmit the incident laser beam and collect the scattered radiation. Laser intensity was mandatorily attenuated to prevent the samples from decomposing.

### First-Principles Calculations

First-principles calculations were carried out using spin-polarized DFT with generalized gradient approximation (GGA) of Perdew-Burke-Ernzerhof (PBE) implemented in VASP code^22,23^. The DFT + U methodology with a value of U = 5.0 eV was used in this work^24^. The valence electronic states were expanded in the basis of plane waves, and the core–valence interaction is represented using the scalar relativistic projector augmented wave (PAW)^25^ approach and a cutoff of 400 eV. More accurate single point electronic structure calculations were further performed using HSE06 functional to obtain the density of states of the Cu_4_Sn_7.5_S_16−*x*_Se_*x*_ system (*x* = 0, 0.5, 1.0, 1.5, 2.0).

### Data availability

All data generated or analyzed during this study are included in this published article (and its Supplementary Information files).

## Results and Discussions

### Compositions and structures

Scanning electron microscopy (S-4800, Hitachi, Japan) was employed to check the homogeneity of the microstructure of the sample (*x* = 1.0). The mapping pictures of four elements (Cu, Sn, S and Se) and EDAX spectrum are shown in Fig. S1. We observed slight segregations for Cu and Se. The average chemical compositions taken from different mappings for each element are presented in Table S1, where the number of moles of S is normalized to 15. Generally, the relative molars of Cu, Sn, S and Se identified are close to those of nominal compositions, only a little deficiency in Se and excess in Sn. These results suggest that the compositions are almost as intended as in the nominal materials.

**Figure 1:**
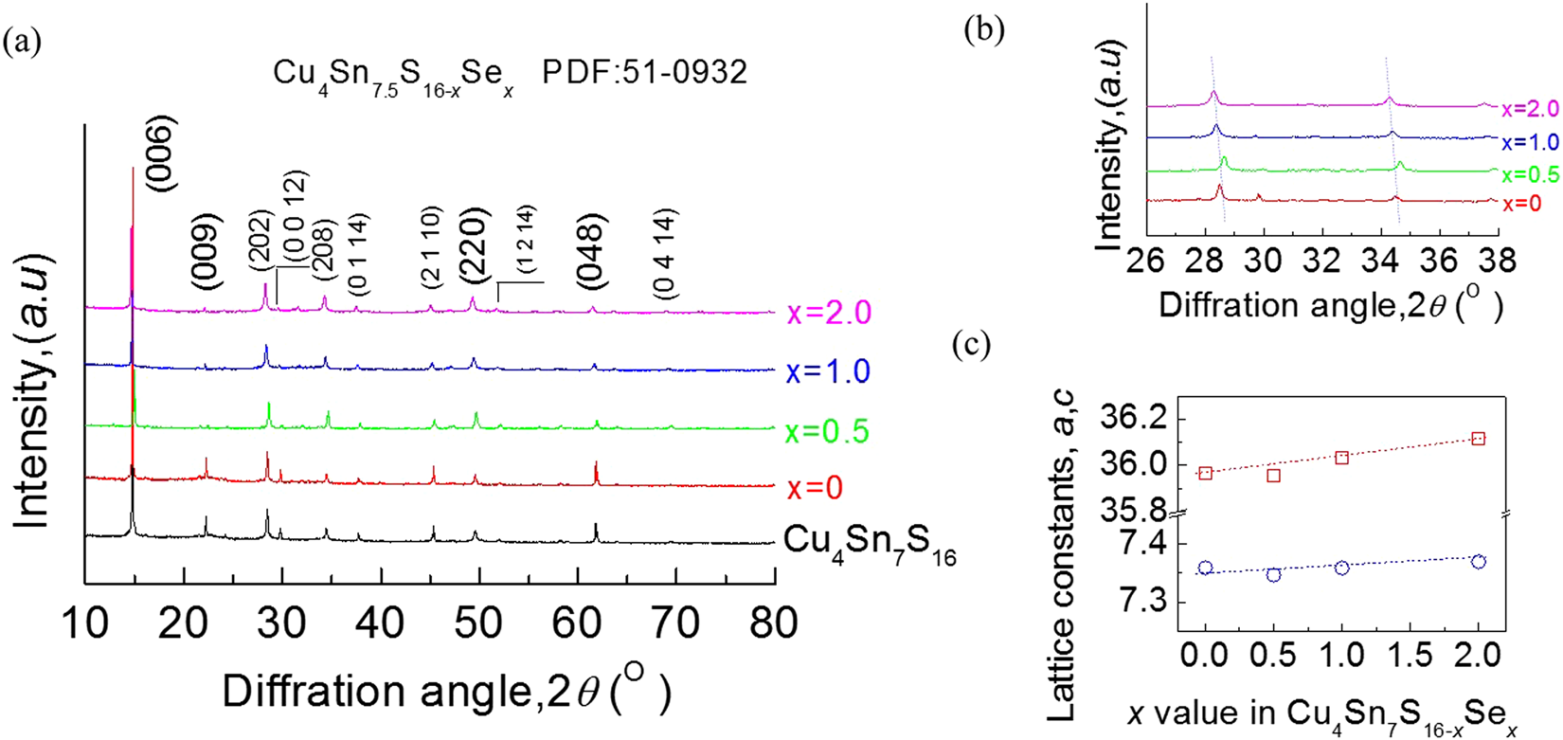
(**a**) X-ray diffraction patterns of Cu_4_Sn_7.5_S_16−*x*_Se_*x*_ (*x* = 0, 0.5, 1.0, 2.0) powders at RT; (**b**) A close-up view with the 2*θ* ranging between 26°–38° (**c**) Lattice constants *a* and *c*, which increase with *x* value increasing, follow the Vegard’s law.

The X-ray diffraction patterns of the powders Cu_4_Sn_7.5_S_16−*x*_Se_*x*_ (*x* = 0, 0.5, 1.0, 2.0) at RT are shown in Fig. 1, where all peak positions are observed the same as those of stoichiometric Cu_4_Sn_7_S_16_ (PDF: 51-0932) with no visible impurity phase identified. The result thus indicates that the synthesized samples are crystallized in a single phase, as shown in Fig. 1a,b is a close-up view of the XRD patterns between 26° to 38°. We observed that the peak positions corresponding to the crystal planes (202) and (208) shift towards low angle, except for the sample at *x* = 0.5, whose peak positions do not follow the trend. The reason is unknown. The possible explanation is that this material contains a serious segregation, which influences its peak positions. Anyhow, the movement in peak positions indicates that the crystal lattice dilates when Se substitutes for S. This dilation is also indicated by linearly increased lattice parameters, *a* and *c*, displayed in Fig. 1c, and can be explained by the larger atomic size of Se (1.18 Ǻ) than that of S (1.04 Ǻ)^26^. As such, the tensile stress might occur.

**Figure 2:**
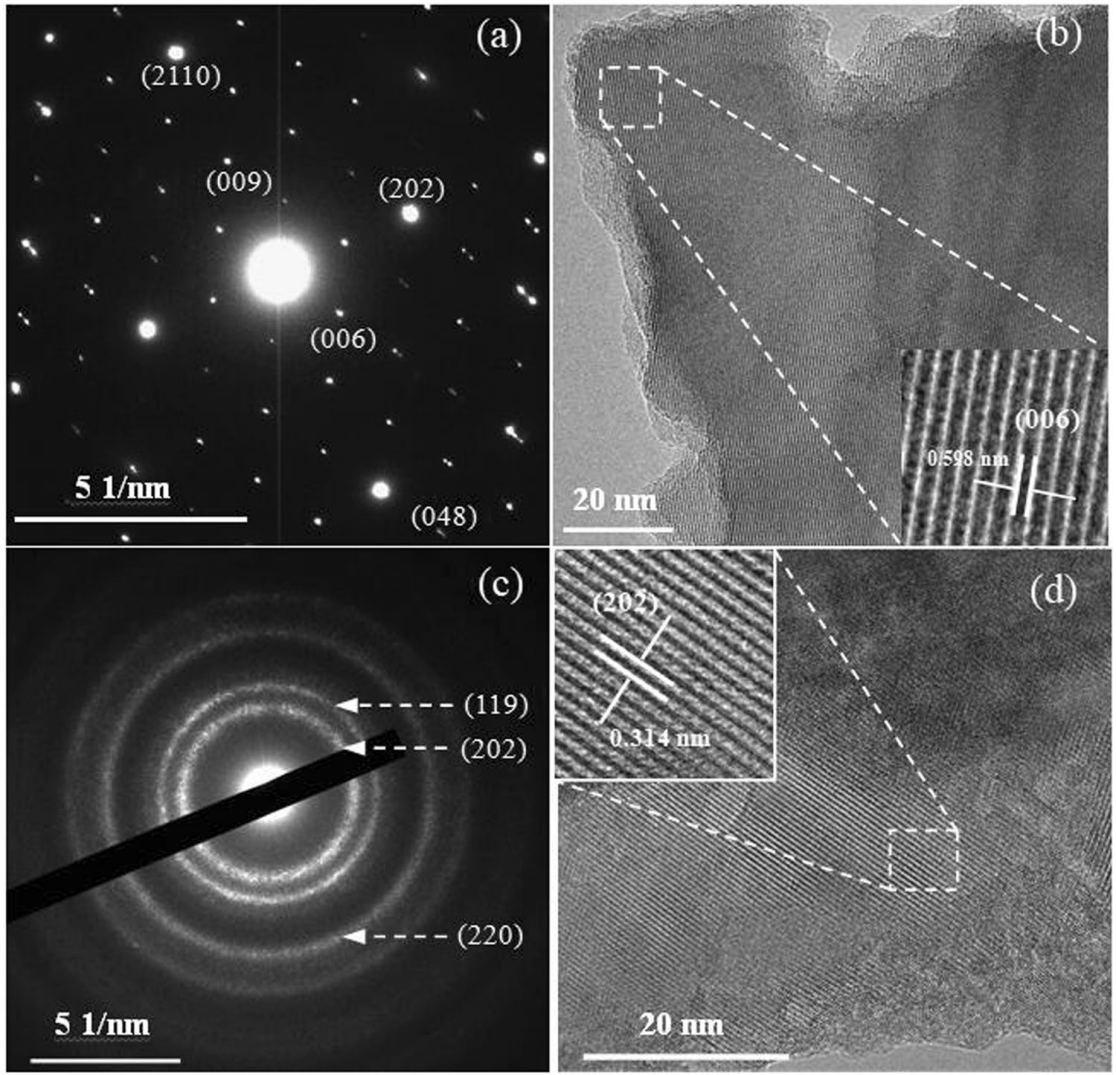
(**a**) A selected area electron diffraction (SAED) pattern for the sample at *x* = 0; (**b**) Its corresponding HRTEM image, an inset is a magnified image, showing that the *d* spacing between (006) crystal planes is about 0.598 nm; (**c**) A SAED pattern for *x* = 2.0; (**d**) Its corresponding HRTEM image, an inset is a magnified image, showing that the *d* spacing between (202) crystal planes is about 0.314 nm.

The microstructure upon an incorporation of Se was observed using high-resolution TEM (HRTEM) for the sample (*x* = 2.0), and that of the Sb-free sample (*x* = 0) are presented for comparison. The results are shown in Fig. 2, where Fig. 2a is a selected area electron diffraction (SAED) pattern for *x* = 0 and Fig. 2b its corresponding HRTEM image. The inset in Fig. 2b is a magnified image, showing that the *d* spacing between (006) crystal planes is about 0.598 nm. Figure 2c is the SAED pattern for *x* = 2.0 and Fig. 2d its corresponding HRTEM image. An inset in Fig. 2d indicates that the *d* spacing between (202) crystal planes is about 0.314 nm. These observations suggest that the Se atoms are almost incorporated into the crystal lattice of Cu_4_Sn_7.5_S_16_ at *x* ≤ 2.0. Besides, both samples exhibit a typical polycrystalline structure, but the grain size in the sample at *x* = 2.0 seems to be smaller than that at *x* = 0.

Upon the incorporation of Se, we believe that Se atoms are most likely settled in the S site rather than in Cu and Sn sites, because Se has the same valence electrons as S. This assumption can be substantiated by the low formation energies (*d*_H_) calculated when Se resides in S sites. Since the *d*_H_ value, which gradually increases from 0.78 (eV) (*x* = 0) to 2.73 (eV) (*x* = 2.0), is much lower than those in the case of Se residing in Cu (8.16 eV) and Sn (5.59 eV) sites when *x* = 0.5. Therefore, we mainly focus on the band structure calculation in the event Se occupies S site.

**Figure 3:**
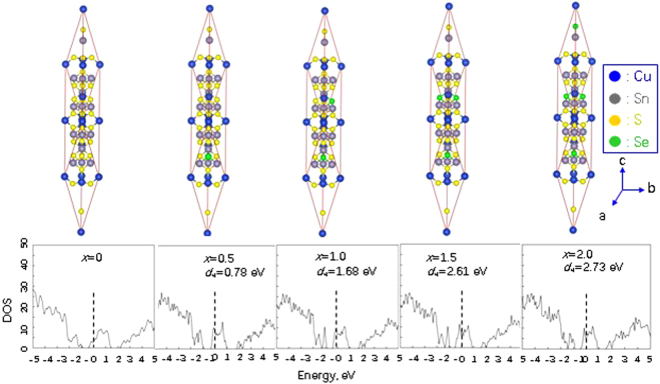
The results from the first principle calculation. (**a**) Upper panel: crystal structures upon the occupation of Se in S sites; (**b**) Lower panel: the density of States (DOS) for Se incorporated Cu_4_Sn_7.5_S_16_. The DOS is more localized in both the valence and conduction bands, and sharply increases in the vicinity of the Fermi level (*F*r) for the samples at *x* = 0.5 and 1.0, exhibiting resonant states. *F*r seems to be pinned in the middle of conduction band, with no visible tendency of movement. The formation energy (*d*_H_) gradually increases from 0.78 (eV) to 2.73 (eV) as Se content increases from *x* = 0 to *x* = 2.0.

Figure 3 shows the density of states (DOS) of the compounds Cu_4_Sn_7.5_S_16−*x*_Se_*x*_ (*x* = 0.5, 1.0, 1.5 and 2.0) pursuant to the first-principles, where the DOS at *x* = 0 is presented for comparison^5^. When Se is incorporated into the S site (Fig. 3), the DOS is more localized in both the valence and conduction bands, and it significantly increases in the vicinity of the Fermi level (*E*_f_) for the samples at *x* = 0.5 and 1.0, exhibiting a behavior of resonant states. At *x* ≥ 1.5, the DOS near the Fermi level decreases. Further, there is a band splitting in the conduction band (CB) between 1.0–1.7 eV, without a tendency of degeneracy. The *E*_f_ seems to be pinned in the middle of CB, with no visible tendency of movement either.

### Transport and TE properties

**Figure 4:**
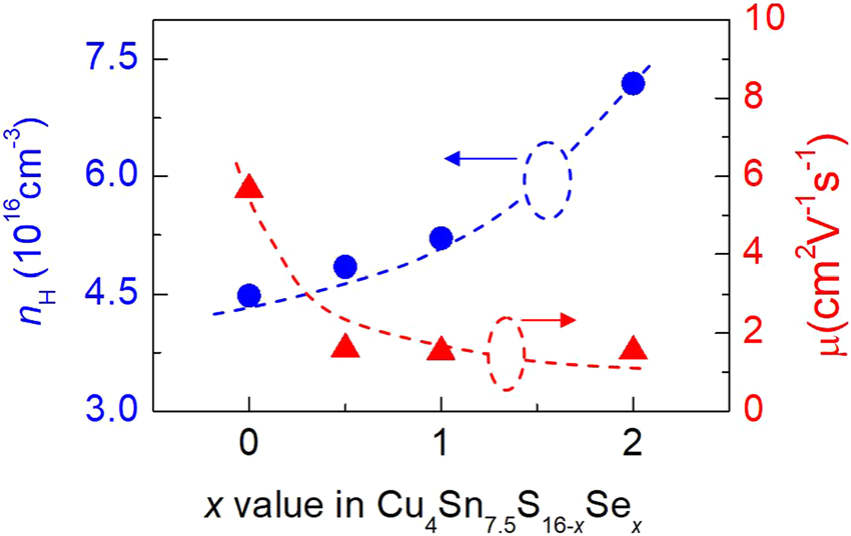
Measured Hall carrier concentration (*n*_H_) and mobility (*μ*) against *x* value in Cu_4_Sn_7.5_S_16−*x*_Se_*x*_.

In order to understand the effects of the band structure modification on the transport and physical properties, we have measured the Hall coefficients of the compounds, and then calculated the Hall carrier concentration (*n*_H_) and mobility (*μ*). The results are indicated in Fig. 4, where we observed visible changes in both *n*_H_ and *μ*. The *n*_H_ value enhances from 4.48 × 10^16^ cm^−3^ (*x* = 0) to 7.18 × 10^16^ cm^−3^ (*x* = 2.0), while the mobility *μ* decreases from 5.66 cm^2^v^−1^s^−1^ to 1.53 cm^2^v^−1^s^−1^. Since the isoelectronic substitution could neither create extra electrons in the compound, nor alter the chemical environment in accordance to the estimation using the valence counting rule^27,28^, therefore, the changes in *n*_H_ and *μ* are mainly attributed to the formation of the conductive network of Cu-Se when Se replaces S.

**Figure 5:**
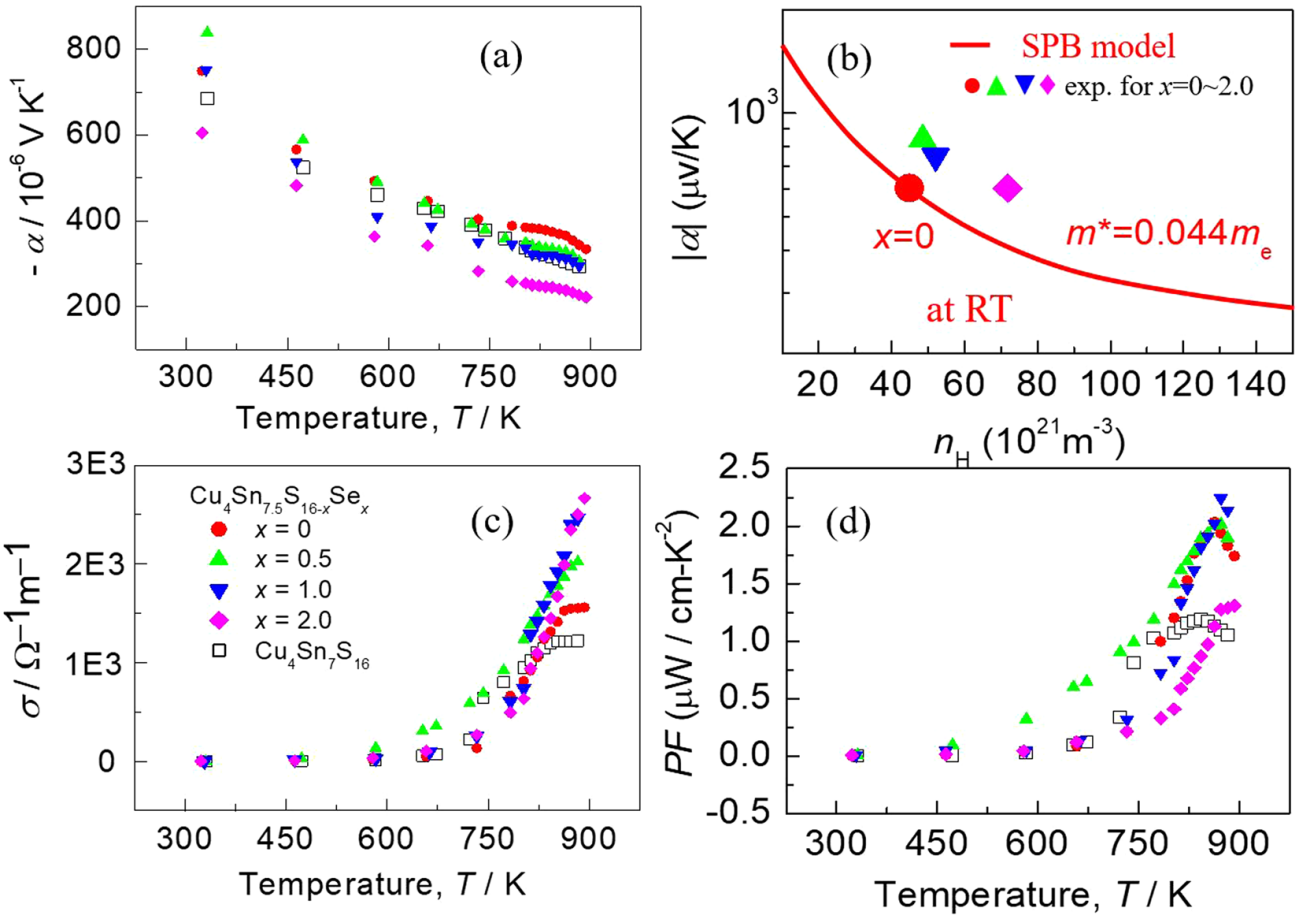
(**a**) Seebeck coefficients (*α*) of the compounds Cu_4_Sn_7.5_S_16−*x*_Se_*x*_ (*x* = 0.5, 1.0 and 2.0), and those for *x* = 0 and Cu_4_Sn_7_S_16_ are presented for comparison; (**b**) Experimentally determined Seebeck coefficients (*α*) at the corresponding Hall carrier concentrations, labeled by . The solid line represents the Pisarenko relation at RT; (**c**) Electrical conductivities (*σ*) as a function of temperature for different materials (*x* values); (**d**) Power factor *PF*, *PF* = *α*^2^*σ*, for different materials (*x* values).

Figure 5 represents the Seebeck coefficient (*α*) and electrical conductivity (*σ*) measured in the temperature range from RT to ~900 K, where those for *x* = 0 and Cu_4_Sn_7_S_16_ are presented for comparison. The *α* value shows no visible decreasing tendency at *x* ≤ 1.0, probably due to the generation of the resonant states near the Fermi level^29^, which increases the relatively effective mass m^*^/*m*. Due to such, the *α* values at *x* = 0.5 and 1.0 are seemingly larger than those at the corresponding carrier concentrations determined by the Pisarenko relation^30^ at RT (indicated by a solid line in Fig. 5b). At ~890 K the *α* values at *x* = 0.5 (1.0) are 306.3 (294.19 μVK^−1^), only 8.3% (11.9%) less than 334.1 (μVK^−1^) at *x* = 0 (Fig. 5a); while the *α* value at *x* = 2.0 is 221.5 μVK^−1^, about 33.7% less. The electrical conductivity (*σ*) (shown in Fig. 5c) increases from 1.57 × 10^3^ Ω^−1^m^−1^ (*x* = 0) to 2.67 × 10^3^ Ω^−1^m^−1^ (*x* = 2.0) at ~890 K as a result of an increased carrier concentration. The power factors *PF*, *PF* = *α*^2^*σ*, are shown in Fig. 5d. At *x* = 1.0 the *PF* reaches the highest (2.24 μW/cm-K^2^) at ~890 K, about 10% higher than that at *x* = 0.

In light of the above measurements, we conclude that the created resonant states near the *E*_f_ and the formation of Cu(Sn)-Se network have a profound impact on the electrical property. They thus play a big role in improving the TE performance.

**Figure 6:**
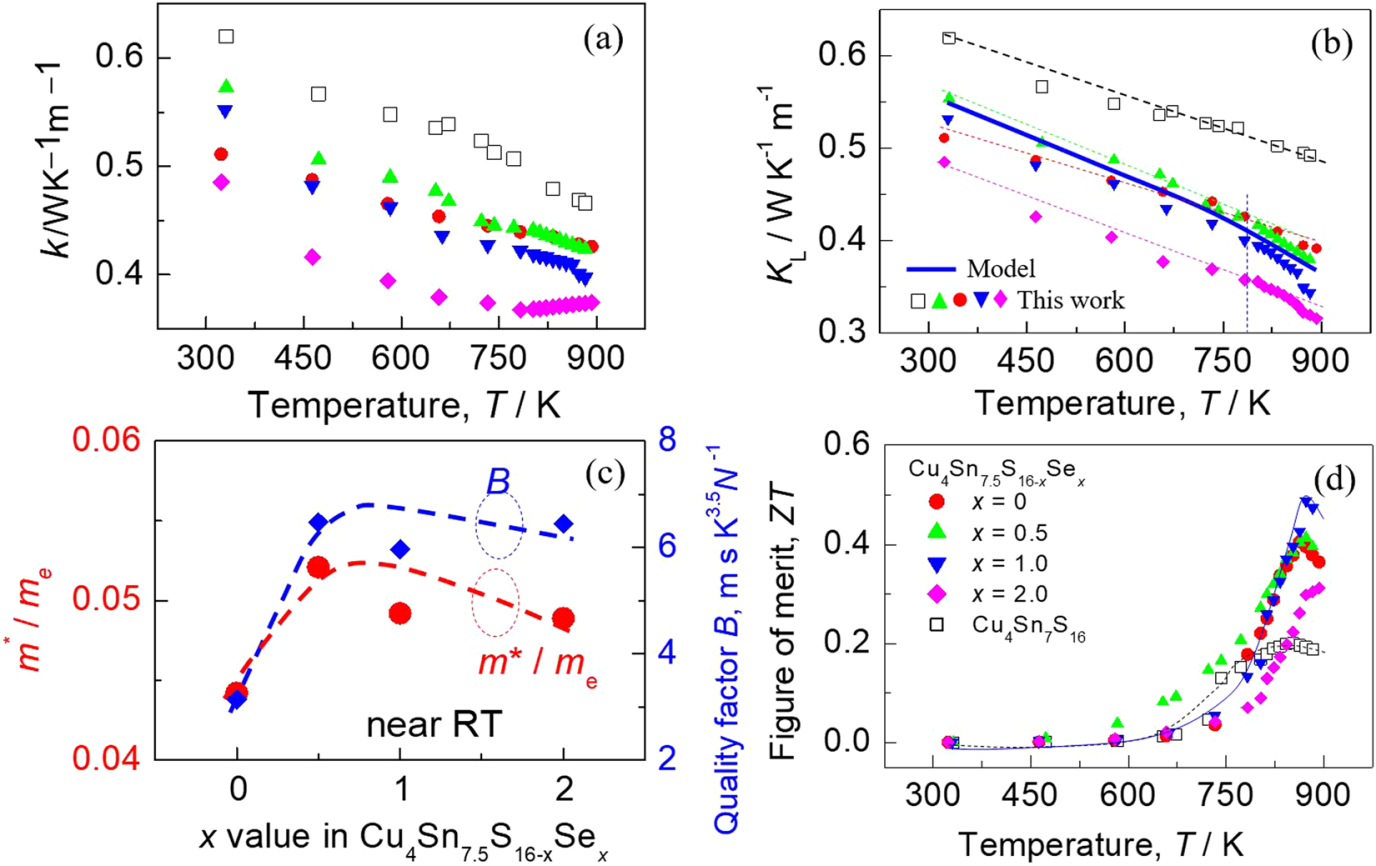
(**a**) Total thermal conductivities (*κ*) as a function of temperature for different materials (*x* values); (**b**) Lattice thermal conductivities as a function of temperature for different materials (*x* values). The solid blue line represents the fitting result using Callaway and Klemens model; (**c**) The values of m^*^/*m*_e_ and quality factor *B* as a function of Se content (*x* value); (**d**) TE Figure of merit (ZT) as a function of temperature for different materials (*x* values).

It is well known that the electronic thermal conductivity *κ*_e_ is determined by the electrical conductivity *σ*, whereas the lattice part *κ*_L_ is substantially independent of the charge carrier system except for, perhaps the scattering of lattice vibrations (phonons) by charge carriers at high carrier densities^21^. At a temperature far above the Debye temperature, all phonon modes are activated, and increased umklapp and lattice disorder and/or phonon-electron scattering would mainly cause the lattice thermal conductivity reduction. Upon Se incorporation, the scattering from introduced lattice disorder along with the umklapp process should play a big role in reducing the lattice contribution. To verify this assumption, we have measured the thermal diffusivities of different samples and obtained thermal conductivities (*κ*), which are presented in Fig. 6a. At a temperature above ~720 K, the *κ* value generally decreases as Se content increases, and at ~890 K it reduces to 0.39 WK^−1^m^−1^ for *x* = 1.0. The composition dependence of lattice parts (*κ*_L_) shares some similarities to total *κ* at *x* ≤ 1.0, and at ~890 K it reduces to 0.34 WK^−1^m^−1^ for *x* = 1.0 (Fig. 6b). Although the lattice part *κ*_L_ decreases as Se content increases, the values of m^*^/*m*_e_ and quality factor *B* (*B* = *μ*_H_(m^*^/*m*_e_)^3/2^*T*^5/2^/*κ*_L_)^21^ increase simultaneously as *x* value increases, until they start to decrease at *x* = ~0.75, as shown in Fig. 6c. This further confirms the importance of the created resonant states near the Fermi level. Combined with the three physical properties (*α*, *σ* and *κ*), we attain the TE figure of merit (ZT), shown in Fig. 6d. At *x* = 1.0 the ZT value reaches the highest (ZT = 0.5) at 873 K, 25% higher compared to ZT = 0.4 at *x* = 0. Although this value is lower that those in other Cu-Sn-S compounds, such as Cu_3_Sn_1.2_S_4_ (ZT = 0.75 at 790 K)^7^ and Co-Cu_2_SnS_3_ (ZT = 0.85 at 723 K)^6^, it still stands out among the highest in the family of Cu_4_Sn_7_S_16_ compounds.

**Figure 7:**
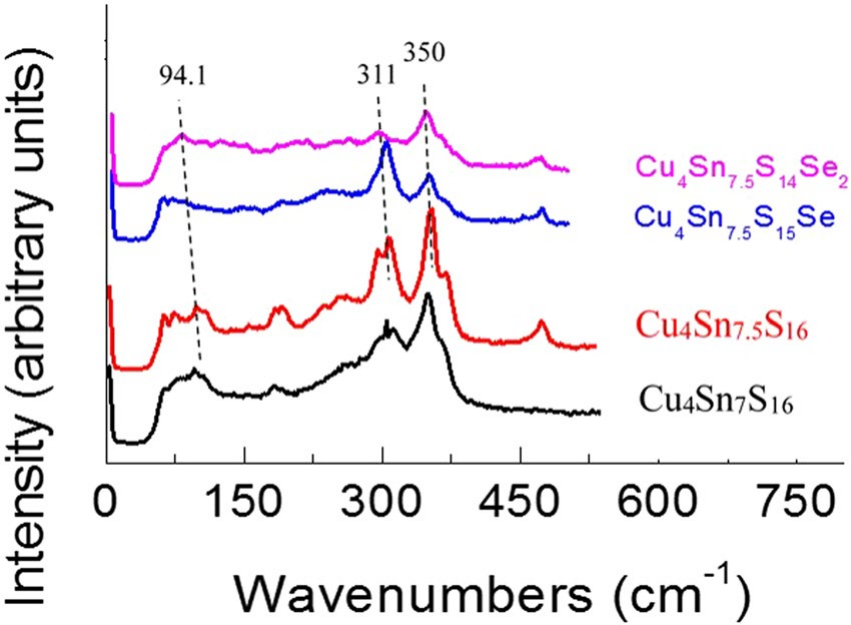
Raman spectra of the Cu_4_Sn_7.5_S_15−*x*_Se_*x*_ (*x* = 1.0 and 2.0) samples, and those of Cu_4_Sn_7_S_16_ and Cu_4_Sn_7.5_S_16_ are just used for comparison. A red shift for the modes at 94.1 cm^−1^, 311 cm^−1^, 350 cm^−1^ is observed with Se content increasing.

**Figure 8:**
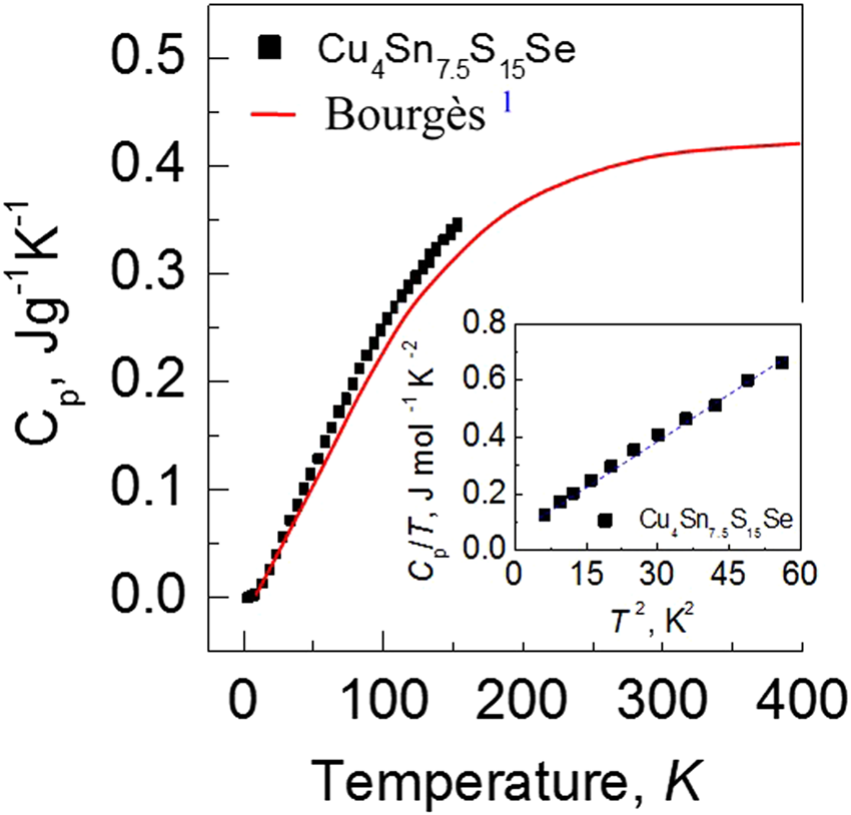
The measured heat capacity *C*_p_ versus *T* at 2.0–153 K for the sample Cu_4_Sn_7.5_S_15_Se. An inset is the *C*_p_/*T* versus *T*^2^ at low temperatures, where the dashed blue line was fitted using a standard Debye model, *C*_p_/*T* = α + β*T*^2^, here α and β are characteristic constants of the materials. The heat capacity *C*_p_ of Cu_4_Sn_7_S_16_ versus *T*, shown in a solid red line, is from Bourgès^1^.

To further verify the increase in phonon scattering upon Se incorporation, we have analyzed the Raman spectra, shown in Fig. 7, where the Raman spectra of Cu_4_Sn_7_S_16_ and Cu_4_Sn_7.5_S_16_^5^ are used for comparison. The features of the presented spectra upon Se incorporation bear an overall resemblance to those of Cu_4_Sn_7_S_16_ and Cu_4_Sn_7.5_S_16_, which suggests that the molecular structures are rather similar upon Se incorporation. However, the main modes at 94.1 cm^−1^, 311 cm^−1^ and 350 cm^−1^ become less pronounced as Se content increases. Meanwhile, the peak position shifts to the low frequency side (red shift) as well. This indicates that the increased structural disorder is a result of the difference in atomic size between Se and S, the changes of bonding between atoms and the presence of tensile stress^31^. Owing to the presence of the tensile stress, the lattice structure expands (Fig. 1c). In addition, the presence of the tensile stress along with the lattice structure dilation is closely related to the creation of the extra conductive paths in the framework and favor the large DOS in both the valence and conduction bands^10,32^. Therefore, the carrier concentration increases. On the other hand, the substitution of Se for S can reduce the Debye temperature^13^ or sound velocity^33^. To confirm this issue, we have measured the heat capacities of the sample Cu_4_Sn_7.5_S_16−*x*_Se_*x*_ (*x* = 1.0) in the temperature range of 2.0–153 K (Fig. 8), and then determined the β value using the relation of *C*_p_/*T* ~ *T*^2^ in the low temperatures, according to Eq. (2). As shown in Fig. 8 and its inset, there is an almost linear relation between *C*_p_/*T* and *T* ^2^. Therefore, the Debye temperatures (Θ_D_) was determined to be 180.8 K. This temperature is much lower than 395 K for the sample Cu_4_Sn_7_S_16_^1^, which indicates the possibility of the extra reduction in lattice part *κ*_L_ at high temperatures^13–16^.

With the help of determined Debye temperatures (Θ_D_ = 180.8 K) of Cu_4_Sn_7.5_S_16−*x*_Se_*x*_ (*x* = 1.0), the dominant effect of structural disorder on the lattice part *κ*_L_ at *T* > Θ_D_ for the sample Cu_4_Sn_7.5_S_16−*x*_Se_*x*_ (*x* = 1.0) can be verified by means of the Callaway and Klemens model^34–37^, assuming that Umklapp and point defect scatterings are the main scattering mechanisms. In this case, the ratio of the modeled lattice thermal conductivity of the crystal with Se substitution for S, *κ*_L_^m^, to the lattice thermal conductivity of the pure crystal, *κ*_L_^p^, is as shown below,

3 $$\frac{{\kappa }_{L}^{m}}{{\kappa }_{L}^{p}}=\frac{{\tan }^{-1}(u)}{u}\,{u}^{2}=\frac{{\pi }^{2}{{\rm{\Theta }}}_{D}{\rm{\Omega }}}{\hslash {\nu }_{m}^{2}}{\kappa }_{L}^{p}{\rm{\Gamma }}$$

where *u* and *Г* are the disorder scaling parameter and the disorder scattering parameter respectively. Here we use the factor *Г* below to predict the *κ*_L_ values for the Cu-Sn-S based chalcogenides^34^,

4 $${\rm{\Gamma }}={\chi }_{i}(1-{\chi }_{i})[{(\frac{{\rm{\Delta }}{M}_{i}}{M})}^{2}+\varepsilon {(\frac{{\rm{\Delta }}{\delta }_{i}}{\delta })}^{2}]$$

where *χ*_i_, Δ*M*_i_/*M* and Δ*δ*_i_/*δ* are the molar fraction of extra Se, the relative change of atomic mass due to the replacement of S by Se, and the local change in lattice parameter. The other related parameters are presented in ref.^5^.

The fitting result using above model is drafted in Fig. 6 as a blue solid curve. Basically, the model enables a good prediction of the temperature dependent lattice thermal conductivity below ~770 K, confirming the dominant effect of the structural disorder upon Se substitution for S. While above ~770 K the estimated *κ*_L_ data is higher than the experimental one, indicating the presence of other phonon scattering mechanism, which is most likely the increased phonon-electron scattering at high temperatures.

## Conclusions

Cu_4_Sn_7.5_S_16_ family of compounds with Se isoelectronic substitution for S have been prepared and their transport and thermoelectric properties have been investigated. The first-principles calculation reveals that upon the incorporation of Se, the Fermi level still pins within the conduction band without any movement tendency. However, the DOS is more localized in both the valence and conduction bands, and increases in the vicinity of the Fermi level (*E*_f_), which indicates the occurrence of resonant states. As such, the effective mass (m^*^/*m*_e_) and quality factor (*B*) increase and thereby profoundly impact the TE performance. Further, there exists another phonon scattering mechanism in the Se-incorporated Cu_4_Sn_7.5_S_16_ system, in addition to the dominant point defect scattering of phonons. This extra mechanism is likely due to the scatterings from the phonon-electron interaction at high temperatures. As a consequence, the TE figure of merit (ZT) enhances by ~25%, if compared to 0.4 for Cu_4_Sn_7.5_S_16_.

## Electronic supplementary material

Supplementary Figure 1 and Table 1
