# Supplementary material for: Improved thermoelectric performance of solid solution Cu4Sn7.5S16 through isoelectronic substitution of Se for S
Source: Sci Rep. 2018 May 29;8:8202. doi: 10.1038/s41598-018-26362-z (PMC5974318; doi:10.1038/s41598-018-26362-z)
Supplement: Supplementary file 1 — Supplementary Figure 1 and Table 1 [file 41598_2018_26362_MOESM1_ESM.pdf]

Supporting Information

Improved thermoelectric performance of solid solution $Cu_4Sn_{7.5}S_{16}$ through isoelectronic substitution of Se for S

Jiaolin Cui,[a*] *Tongtong He,*[a] *Zhongkang Han,*[b*] *Xianglian Liu* [a], *Zhengliang Du* [a]

[a] School of Materials & Chemical Engineering, Ningbo University of Technology, Ningbo, 315211, China

[b] Shanghai Institute of Applied Physics, Chinese Academy of Sciences, 201800, China

Corresponding author's contact information:

Jiaolin Cui:

School of Materials & Chemical Engineering, Ningbo University of Technology, Ningbo 315211, China

E-mail: cuijl@nbut.edu.cn

Tel: 86-0574-87080504, Fax: 86-0574-87080504

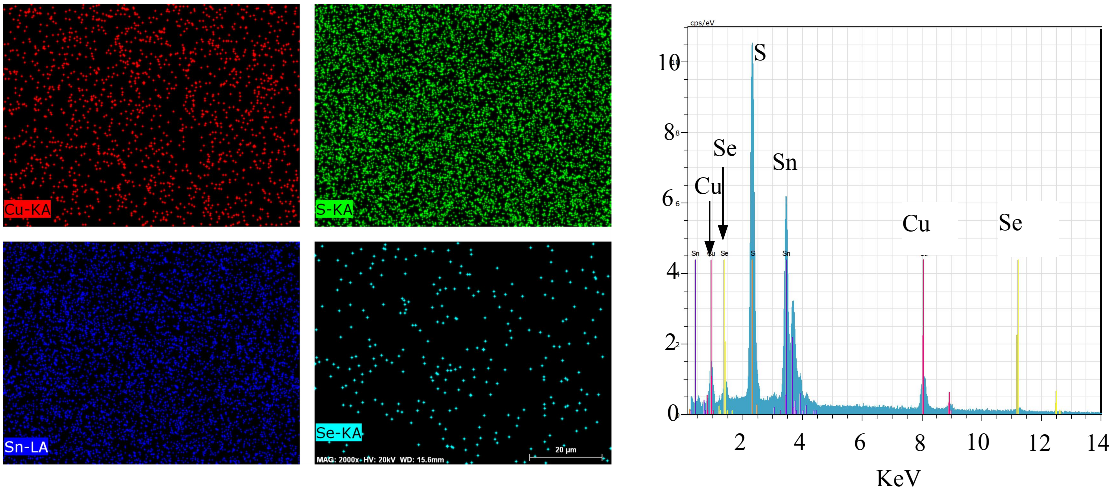

Fig.S1 Left panel: EPMA mapping of four elements, Cu, S, Sn and Se on polished $Cu_4Sn_{7.5}S_{16-x}Se$ (x=1.0) surface; Right panel: EDAX analysis of the chemical compositions.

Table S1 The chemical compositions of the sample $Cu_4Sn_{7.5}S_{15}Se$ determined using EDAX.

Composition	Cu	Sn	Se	S
$Cu_4Sn_{7.5}S_{15}Se$	3.96	7.55	0.94	15.0
